# Experimental Investigation of Electro-Mechanical Behavior of Silver-Coated Teflon Fabric-Reinforced Nafion Ionic Polymer Metal Composite with Carbon Nanotubes and Graphene Nanoparticles

**DOI:** 10.3390/polym14245497

**Published:** 2022-12-15

**Authors:** Ch Sridhar Yesaswi, Santosh Kumar Sahu, P S Rama Sreekanth

**Affiliations:** School of Mechanical Engineering, VIT-AP University, Besides AP Secretariat, Amaravati 522237, Andhra Pradesh, India

**Keywords:** Nafion, ionic polymer metal composites, CNT, graphene

## Abstract

Ionic Polymer Metal Composites (IPMCs) are in high demand owing to the ongoing advancements in technology for various applications. New fabrication techniques and a quick retort towards the applied load are the significant reasons for considering IPMCs in smart devices. Here, a Teflon fabric-reinforced Nafion (TFRN) membrane is used to create an IPMC. The materials employed as electrodes are silver and nanofillers. The basement membrane, Nafion 438 (N-438), is sandwiched between the electrodes using a chemical decomposition technique. Subsequently, the electromechanical properties (actuation) of the membrane are tested. The micro and molecular structure of the IPMC membrane coated with Silver (Ag), Ag-Carbon nanotubes (CNTs), and Ag-Graphene nanoparticles samples are examined with the help of SEM and X-ray diffraction (XRD). The membrane scratch test is carried out to evaluate the abrasion and wear resistance of the membrane. The lowest coefficient of friction is shown by N438 + Ag + Graphene (0.05), which increased by 300% when compared to a pure N438 membrane. The hydration and tip deflection test were also performed to understand the water uptake percentage. At 90 °C, the highest water uptake was observed for N438 + Ag + Graphene (0.05), which decreased by 60, 42, 23, 14 and 26% when compared to N438, N438 + Ag, N438 + Ag + CNT (0.01), N438 + Ag + CNT (0.05) and N438 + Ag + Graphene (0.01), respectively. A proportional relationship between hydration level and tip deflection is observed and the highest bending performance is observed for the N438 + Ag + Graphene (0.05) membrane.

## 1. Introduction

Ionic polymer metal composites (IPMCs) are categorized as Electro-active Polymers (EAP), which have an inbuilt tendency towards sensing and actuation [1]. Performance is greatly affected with minimal electric potential. These materials have distinctive inherent qualities, such as high sensitivity, polarity, and biocompatibility with pliable manufacturing, which enable them to function in both dry and moist situations [2]; therefore, these materials have had an impact in the medical field [3] and in engineering applications [4]. The addition of appropriate nanofillers into polymeric materials can further enhance functionality by improving thermal [5,6,7], mechanical [8,9,10], rheological [11,12] and tribological [13,14,15] properties.

In recent decades, at various conditions and constraints, the nature of Nafion with respect to the ion exchange rate has been analyzed [16]. Nafion has backbone chains in the hydrophobic semi-crystalline region, which make it more structurally stable. Side chains are present in sulphonic acid groups and proton conductivity is observed in amorphous regions. Nafion is chemically stable due to the presence of a perfluorinated vinyl polyether (side chain) with sulphonic acid groups and with Polytetrafluoroethylene (PTFE). The transport properties of the ions and the mechanical characteristics of the Nafion depend on parameters such as hydrated ions [17], the ambient temperature of the membrane, and the humidity percentage in the air. Generally, based on the water content in the membrane, the transport properties are affected [18]. Cations with a larger radius are recommended so as to reduce chain movement since they offer greater resistance to distortion [19].

For pure Nafion, XRD peaks are observed at 19° and 40° [20], which indicates the crystalline nature of the Nafion; these peaks are associated with the amorphous region of a sulfonated polyimide. For other Nafion composites, at different weight percentages (20%, 40%, 50%, 60% and 80%), XRD peaks are observed, and these correspond with the crystalline and amorphous regions of nanocomposite materials [21]. Pourzare et al. [22] worked on the fabrication of Nafion with magnetic nanoparticles as fillers. XRD patterns of Co_3_O_4_ coated with SiO_2_ are observed with a weak band around 18° to 29° due to the development of amorphous silica encompassing Co_3_O_4_ [23]. From the indices, it can be noted that the Nafion with nanoparticles was completely pure spinal Co_3_O_4_ [24]. Uzundurukan et al. [25] synthesized CNT-Graphene assisted Platinum-Silver, Platinum and, silver catalysts when analyzing the fuel cell performance (sodium borohydride/hydrogen peroxide (NaBH_4_/H_2_O_2_). For CNT-Graphene, broad peaks are observed clearly at 25.7° [26], whereas for other samples, five peaks are observed at 2θ = 38.11°, 44.30°, 64.55°, 77.57° and 81.84° with indices values (111), (200), (220), (311) and (222) planes, respectively (corresponded to the planes of the face-centered cubic structure of silver and silver alloys). Similarly, for Platinum-Silver/CNT-Graphene, FCC peaks and the indices values are observed as (111), (200), (220), (311), and (222) at 2θ = 38.09°, 44.35°, 64.56°, 77.47°, and 81.77°, respectively. The difference in the fcc phases of PtAu/CNT-G and Au/CNT-G indicates lattice expansion, and part of the silver atoms could be incorporated into a platinum lattice; the diffraction peaks of Pt at 39.81° (111), 46.15° (200), and 67.37° (220) show Pt with crystallized nature in an fcc structure [27]. Zhang et al. [28] investigated the impact of hydration on the transportation of water molecules and hydronium ions on N117 at different temperatures. Hydration levels played a significant role in the phase separation of the membrane. Safronova et al. [29] increased the water uptake and conductivity of the Nafion by performing ultra-sonication for 30–40 min. Ye et al. [30] investigated the performance of the Nafion/lignin composite membrane. Nafion 112 demonstrated the highest water uptake of 10.2%, and the remaining composite membranes revealed uptakes of 6.2%, 4.2%, and 10%, indicating that the composite membrane performs better. Marland et al. [31] investigated the Nafion adhesion strength by performing a scratch test using a Nanoindenter G220. Optical inspection over the scratches showed that the membrane underwent plastic deformation followed by buckling and failure of the layer. Profilometry was selected to pinpoint the precise moment of failure. Biswal et al. [32] fabricated a multilayered structure of the IPMC actuator and investigated the bending response at different input voltages. By increasing the number of layers over the base Nafion -117, actuation of the IPMC actuator was increased by nearly 30% (when four layers are added). Luqman et al. [33] identified the deflection behavior of the IPMC actuator. Deflection has been observed in both negative and positive voltages (−4 to +4). Deflection is observed more frequently during positive voltages rather than negative voltages, which is due to hysteresis. Minimum deflection of the SPVC–PTA–Pt polymer soft actuator is around 6 mm, whereas maximum deflection is 15.3 mm at 4 V.

Clearly, the literature indicates that no work was carried out on either coated (nano) or uncoated N438 (Teflon Fabric Reinforced Nafion (TFRN)) membranes. Nonetheless, the performance (as actuators and sensors) of the Nafion can be enhanced by the addition of nanoparticles over the Nafion membrane. In the current work, electromechanical actuation as well as determination of the morphological properties of the Nafion 438 membrane (coated with Ag, Ag-CNT (CNT with 0.01 and 0.05 *w*/*v* ratio) and Ag-graphene (graphene with 0.01 and 0.05 *w*/*v* ratio) are novel contributions to the relevant field.

## 2. Materials and Methods

### 2.1. Materials

#### 2.1.1. Nafion 438

The N438 membrane was acquired from the Power Nafion^TM^ shop. It has greater chemical resistance with polytetrafluoroethylene (PTFE) monofilament reinforcement with dimensions of 300 × 300 × 0.320 mm^3^. This is also known as Teflon Fabric Reinforced Membrane (TFRN). Uniformity in expansion and less leakage are its notable features.

#### 2.1.2. Silver Nitrate (AgNO_3_)

Silver nitrate, which is less soluble in compounds with ether groups and more soluble in aqueous ammonia, was purchased from Fisher Scientific Co. in crystalline form, with more than 98% purity and with a melting point of greater than 200 °C.

#### 2.1.3. Carbon Nano-Tubes (CNTs)

CNT powder was purchased from M/s Shenzhen Nanotech Port Co., Ltd., Shenzhen, China, with 95% purity, with a density of 2.16 g/cm^3^. The length of the particle is approximately between 5 and 10 µm, whereas the outer diameter of the particle is 40 to 60 micrometers. To improve the properties of the CNT, chemical treatment was carried out, as reported by Sreekanth et al. [34].

#### 2.1.4. Graphene

**Figure 1:**
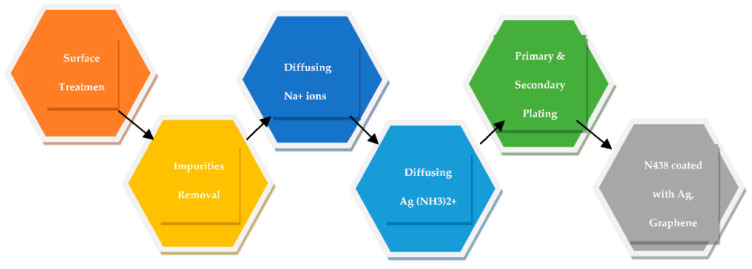
Fabrication Steps.

Graphene (2D crystalline structure) was purchased from Platonic Nanotech with more than 98% purity and a thickness of 2 to 10 nm. It has a honeycomb lattice and multiple layers of carbon. Table 1 represents its elemental percentage. For the fabrication of Ionic Polymer Metal Composites, a chemical plating technique was used [35]. Figure 1 shows the steps involved in fabrication. During surface treatment, a P800 grinding sheet is used to increase the surface area and to remove the impurities, and the material is boiled in a 2N HCL solution. Further, to diffuse the Na+ ions, samples are placed in the NaOH solution and stirred for a certain period of time. Diamine silver hydroxide is prepared by diffusing Ag(NH3)2+. Both primary and secondary plating is carried out for the deposition of particles, and to remove the loosely deposited particles, ultrasonication is performed.

### 2.2. Methods

#### 2.2.1. Scanning Electron Microscopy (SEM)

Using JSM-IT500 InTouchScope™/Jeol Scanning Electron Microscopy (SEM, Tokyo, Japan), the surface morphology of the specimens was studied at various magnifications. The experiment was performed on a sample size of 5 mm × 10 mm at 30 kV with accelerating voltage.

#### 2.2.2. Energy Dispersive Spectroscopy (EDS)

Using a JCM-6000 PLUS/Jeol (Tokyo, Japan), elemental characterization was carried out on all the samples (sample size −5 mm × 10 mm) with a 512 × 384-pixel resolution in the energy range of 0–20 keV and 15 kV.

#### 2.2.3. X-ray Diffraction (XRD)

To further identify the chemical composition and structure of the prepared samples, an XRD analysis was performed using S-3700N—Hitachi (at 40 kV and 30 mA) (Tokyo, Japan). The scan range is 10.000 to 80.000, with a scan speed of 6.0000 (deg/min).

#### 2.2.4. Scratch Testing

The scratch test was performed on a DUCOM Scratch tester TR-101-IAS, which has a load of up to 200 N (Tokyo, Japan). The scratching speed was 10 mm/min with a loading speed of 2 and 3 N/min. Five readings were taken on each sample for higher accuracy and to develop a better understanding.

#### 2.2.5. Hydration Level and Temperature

Water uptake of the IPMC membranes (all the samples) was carried out based on the equation below. Water uptake is defined as the ratio of the absorbed water to the mass of the sample in the dry state.

M(T) = [W_wet_ − W_dry_]/W_dry_

where, M(T)—water uptake/update or hydration mass; T—temperature; W_wet_—weight of the sample in wet state; W_dry_—weight of the sample in dry state (the sample was measured before immersing in water; to ensure there is no water absorption, the dry samples were heated in vacuum oven at 90 °C for 5 h).

The test is performed over the samples after soaking them for 1 h in a hot bath from 30 to 90 °C with a temperature interval of 15 °C.

## 3. Results and Discussion

### 3.1. SEM Analysis

**Figure 2:**
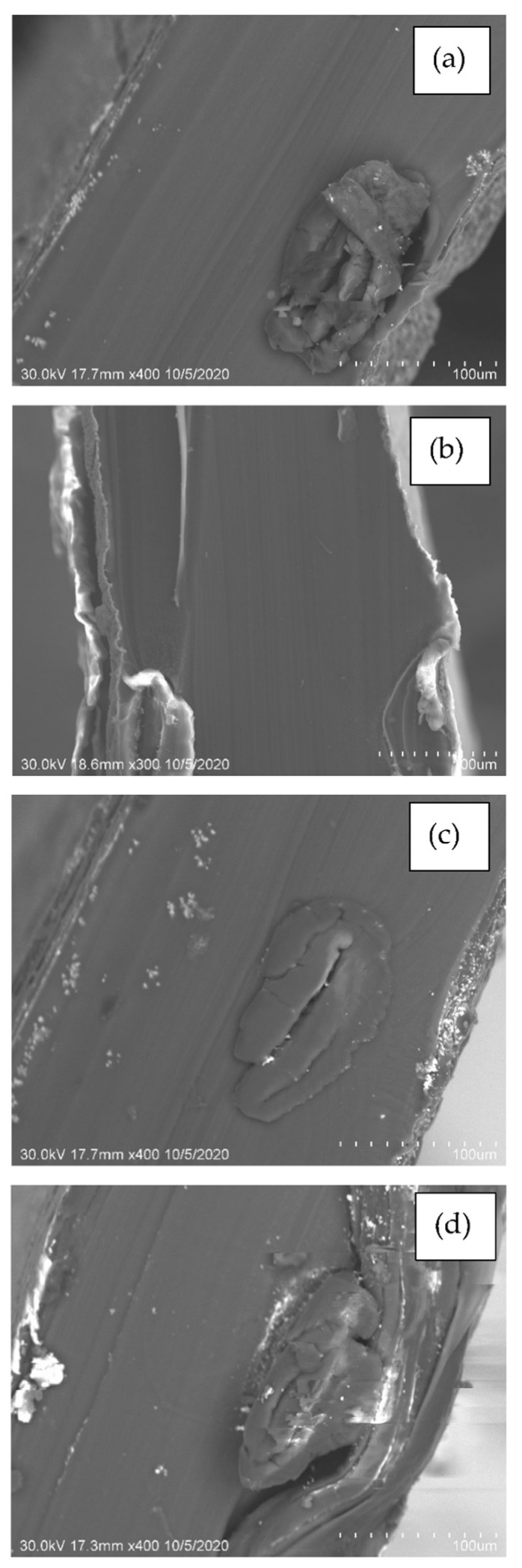
SEM images of (**a**) N438, (**b**) N438 coated with Ag, (**c**) N438 coated with Ag-CNT and, (**d**) N438 coated with Ag-Graphene Energy dispersive spectroscopy (EDS) analysis.

An SEM analysis was carried out for all the samples and the respective images are shown in Figure 2. On the anodic side, the development of bacteria (aerobic and anaerobic) was observed on the N438 membrane, due to environmental effects [36]. Due to the presence of biofilms or bacteria on the membrane, proton movement and oxygen diffusion are expected. The reason for biofilm development is yet to be identified. In contrast, the presence of Ag, Ag-CNT, and Ag-Graphene particles are observed on the membrane as well as the presence of binary oxides on the N438 membrane surface. Nevertheless, no cracks were observed on any of the sample surfaces, which is in agreement with the results of Chen et al. [37]. The fundamental cause of this is the carbon-supported catalyst and the thickness of the membrane, which resists the development of cracks [38].

### 3.2. Energy Dispersive Spectroscopy (EDS) Analysis

**Figure 3:**
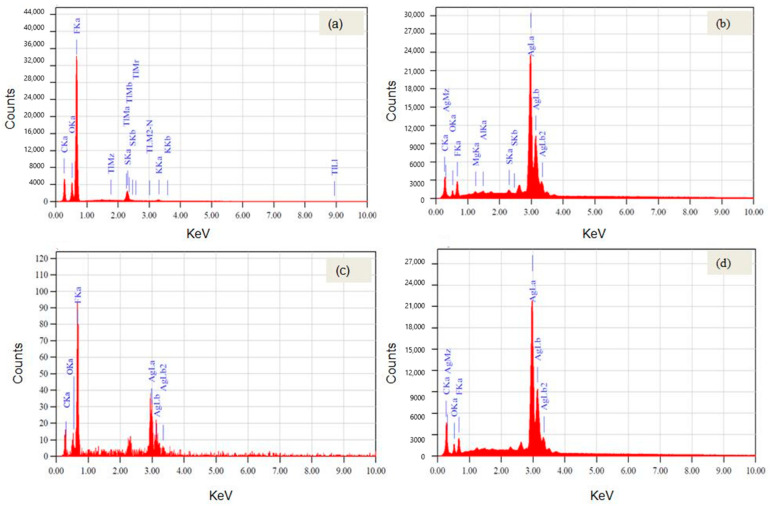
Elemental composition of (**a**) Nafion, (**b**) Nafion + Ag, (**c**) Nafion + Ag + CNT and (**d**) Nafion + Ag + Graphene.

Figure 3 shows the EDS analysis graph. Elements such as oxygen (O), carbon (C), fluorine (F), potassium (K), thallium (Tl), and sulfur are identified in the TFRN membrane. On the remaining samples, Ag_2_O is observed due to the Ag coating. For N438 coated with Ag, the highest mass percentage of silver oxide is observed (91.87%). More cations are observed for N438 coated with Graphene and CNT, which shows more electrons than protons for a net positive charge. In contrast, for N438 coated with Ag, Aluminum was observed because of reactions with sulfur atoms [6]. Table 2 represents the mass percentage of the elements for different samples.

### 3.3. XRD

**Figure 4:**
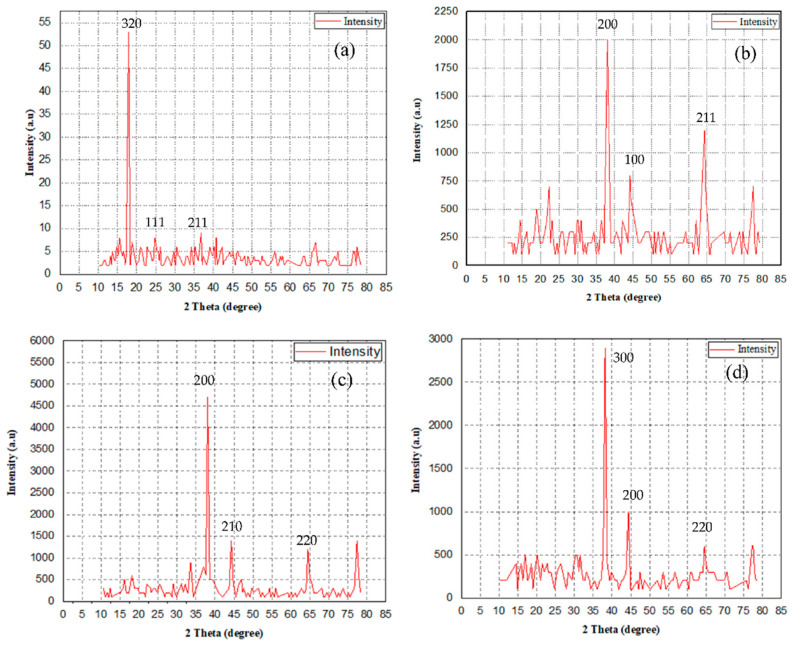
XRD patterns—(**a**) Nafion, (**b**) Nafion + Ag, (**c**) Nafion + Ag + CNT and (**d**) Nafion + Ag + Graphene.

Figure 4 shows the XRD patterns of the Nafion, Nafion + Ag, Nafion + Ag + CNT and Nafion + Ag + Graphene composite membranes samples. From Figure 4a, the XRD pattern of Nafion shows a broad peak at 17.93°, 24.75°, and 36.80°, indicating the crystalline nature of the polymer; these are due to the sulfonated polyimide [39]. Similarly, for Nafion + Ag (Figure 4b), peaks are observed at 38.05°, 44.18°, and 64.37°. In contrast, for the Nafion + Ag + CNT (Figure 4c) and Nafion + Ag + Graphene (Figure 4d) samples, the highest peaks are observed at 38.187°, 44.36°, and 64.52°; 38.06°, 44.30°, and 77.41°. The reasons for smaller and broader peaks (in Figure 4b–d) are the crystalline nature of polymer and nanoparticles associated with the amorphous and crystalline regions of the composite membrane [40].

### 3.4. Scratch Test

**Figure 5:**
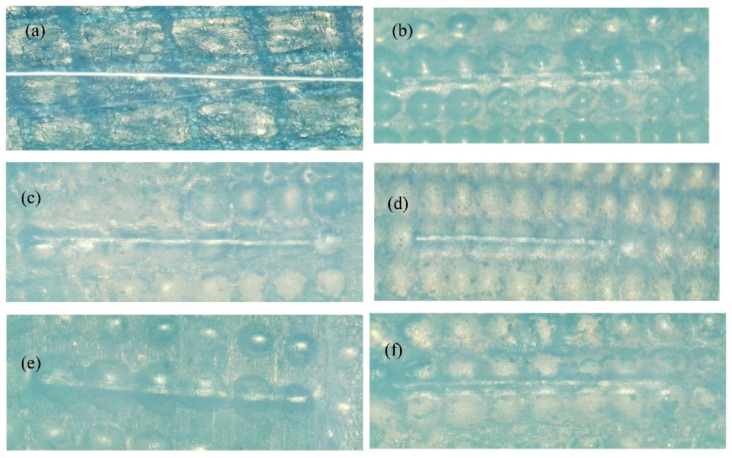
Scratch Test over the different samples (**a**) Nafion, (**b**) Nafion + Ag, (**c**) Nafion + Ag + CNT (0.01), (**d**) Nafion + Ag + CNT (0.05), (**e**) Nafion + Ag + Graphene (0.01) and (**f**) Nafion + Ag + Graphene (0.05).

**Figure 6:**
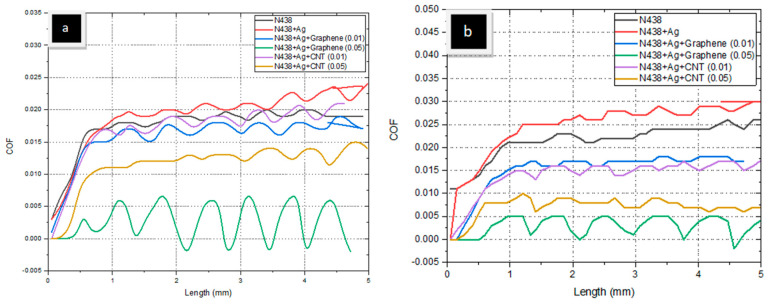
Coefficient of Friction at (**a**) 2N and (**b**) 3N.

**Figure 7:**
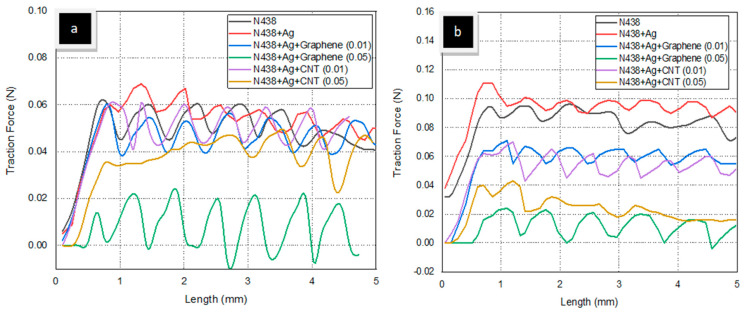
Traction Force at (**a**) 2N and (**b**) 3N.

The scratch test was performed on all the samples at two different loads (2N and 3N). Figure 5 represents the scratch over the surface membrane. Figure 6a,b represent the relationship between the scratch length and the coefficient of friction (COF), and Figure 7a,b represent the traction force over N438, N438 + Ag, N438 + Ag + Graphene (0.01), N438 + Ag + Graphene (0.05), N438 + Ag + CNT (0.01) and N438 + Ag + CNT (0.05) at different loads. From the above Figures (Figure 6a,b), N438 + Ag demonstrated the highest COF, followed by pure Nafion; N438 + Ag + Graphene (0.05) has the lowest COF. For N438 + Ag, COF started varying from 0.020 to 0.025 because, after applying the load through the indenter, its COF increased linearly, followed by a nonlinear trend due to the sample’s surface. From this, it was understood that the indenter can cut the material surface easily; in contrast, for N438 + Ag + Graphene (0.05) and N438 + Ag + CNT (0.05), due to the addition of fillers, the matrix surface was strengthened and demonstrated less COF and smooth curves. The reason for this high bonding between the matrix and fiber is due to the addition of nano-materials [41], which results in scratch resistance. The scratch image over the Nafion (N438) membrane is due to the plowing frictional effect [42]. Similarly, N438 + Ag is shown to have the highest traction force, and N438 + Ag + Graphene (0.05) is shown to have the lowest.

### 3.5. Hydration Test

**Figure 8:**
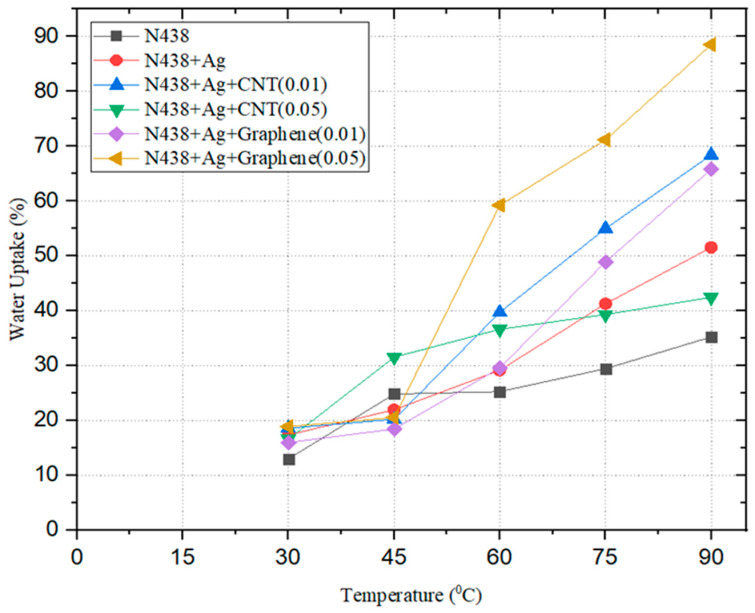
Water uptake % vs. Temperature.

The water uptake of the N438, N438 + Ag, N438 + Ag + Graphene (0.01), N438 + Ag + Graphene (0.05), N438 + Ag + CNT (0.01), and N438 + Ag + CNT (0.05) samples is taken into consideration at different temperatures. The nanostructure of the composite membrane and the temperature play a crucial role in the water uptake of the composite membranes [43]. Before immersion in water, the weights of the dry samples were measured. Then, each sample was immersed in water and soaked for 1 h from 30 to 90 °C, and the weight of the respective sample was measured (wet samples). Table 3 represents the Wdry of the samples. Figure 8 shows the water uptake percentage increase with hydration temperature. The simplified values from the figure are shown in Table 4. Graphene’s high-water uptake is due to its tendency to adsorb and the water uptake for Nafion agrees with the results in the literature [44]. The main reason is oxygen-containing functional groups on its hydrophilic surface [45]. Similarly, CNT shows that a higher water uptake is due to the presence of hydrophilic groups [46]. The reason for Graphene having a higher value compared with CNT is due to its higher specific surface area.

### 3.6. Tip Deflection and Tip Force Measurement of the IPMC Membranes

Under the fixed free configuration of the membrane/sample, the tip force and tip deflection of the N438 + Ag, N438 + Ag + Graphene (0.01), N438 + Ag + Graphene (0.05), N438 + Ag + CNT (0.01) and N438 + Ag + CNT (0.05) samples are taken into consideration at room temperatures and four different input voltages.

**Figure 9:**
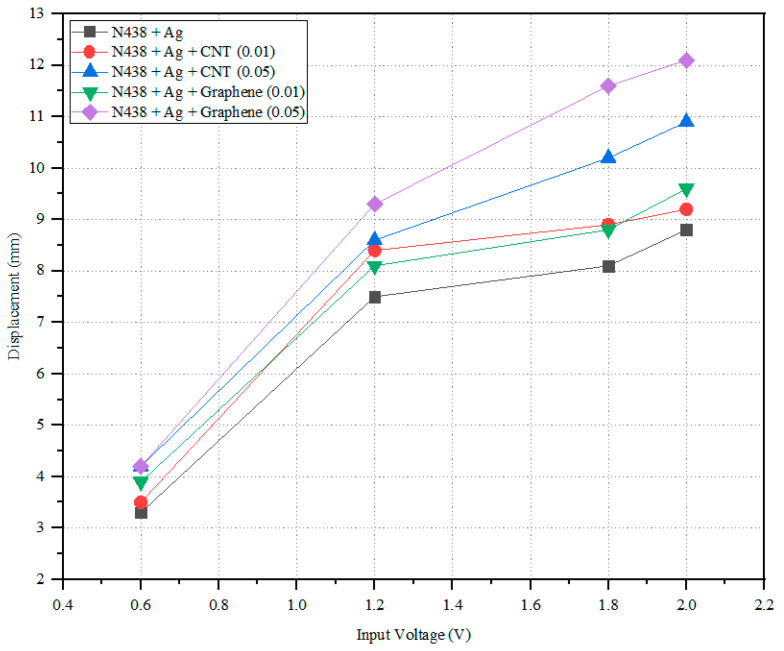
Input voltage vs. displacement.

**Figure 10:**
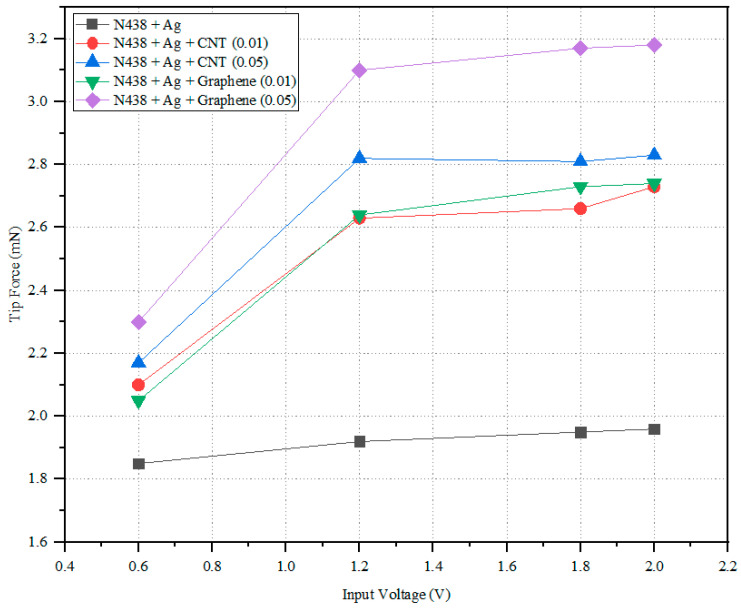
Input voltage vs. tip force.

Figure 9 represents the input voltage vs. load for various samples, and the simplified results are shown in Table 5. It is understood that Graphene with a higher concentration (0.05) demonstrated the highest displacement of 12.1 mm, followed by CNT (0.05) with 10.9 mm. The lowest level is observed in the N438 + Ag and N438 + Ag + CNT (0.01) composite membranes. This indicates that with minimum potential, N438 + Ag + Graphene (0.05) has a higher actuation ability when compared to the remaining composite membranes. The reason for this is the higher conductivity rate of the Graphene and the difference in electrical conductivity is due to the microstructure of CNT and Graphene [47]. Graphene is a 2D single sheet with an allotrope of carbon atoms. In contrast, CNT has (1D) a honeycomb structure or a grid of carbon atoms which are unaffected [48]. The cause for the smaller difference in the conductivity between Graphene and CNT is that out of four electrons in the outer shell of the carbon atom, three electrons lead to covalent bonding, and the remaining electron tends to move freely, which leads to good electrical conduction. Figure 10 shows the input voltage vs. tip force. Table 6 represents the developed tip force of the composite membranes at different input voltages. From the graph, N438 + Ag + Graphene (0.05) shows the highest tip force of 3.18 Mn, and N438 + Ag (1.85 mN) has the lowest, which could be due to the microstructure of CNT and Graphene. This shows that the N438 + Ag + Graphene (0.05) membrane has a high lifting capacity compared to the remaining composite membranes.

## 4. Conclusions

In the present work, TFRN membranes coated with Ag, Ag-CNT and Ag-Graphene at different ratios have been studied and the morphology of the membranes has been discussed. Additionally, their electro-mechanical behavior has also been analyzed. Based on the investigation of the membranes, the following conclusions were drawn and summarized:

- The highest scratch resistance and the lowest COF were observed in the N438 + Ag + Graphene (0.05) and N438 + Ag + CNT (0.05) membranes, which is because the fillers develop high bonding between the matrix and fiber.
- Graphene coating demonstrated the highest water uptake due to the presence of functional groups on its surface (hydrophilic), and because it stores water molecules effectively.
- Tip force and tip deflection were shown to be highest for Graphene (0.05), immediately followed by CNT (0.05) with a small difference, due to the presence of free electron mobility. This indicates that both composites can be used in the application of sensors and actuators.

## Figures and Tables

**Table 1:** Elemental percentage.

Elements	Carbon	Oxygen	Hydrogen	Sulfur	Ash
Percentage	81	11	7	<0.8	<0.2

**Table 2:** Mass percentage of the elements for different samples.

Elements	N438	N438 + Ag	N438 + Ag + CNT	N438 + Ag + Graphene
C	21	7.24	5	4.8
O	9.2	6.31	3.6	7.4
F	5.17	1.43	30.6	1.5
S	5.10	0	0	0.53
K	0.76	0	0	0
Tl	12.15	0	0	0
Ag	0	85.02	60.8	85.5
Mg	0	0	0	0.13
Al	0	0	0	0.18

**Table 3:** Weight of the Samples before immersing in hot water.

Sample	N438	N438 + Ag	N438 + Ag + CNT (0.01)	N438 + Ag + CNT (0.05)	N438 + Ag + Graphene (0.01)	N438 + Ag + Graphene (0.05)
W_dry_ (gm)	0.0693	0.0652	0.0544	0.0669	0.0683	0.0721

**Table 4:** Weight of the Samples after immersion in hot water and their respective water uptake.

Sample	Temperature °C	30	45	60	75	90
N438	W_wet_ (gm)	0.0783 ± 0.0012	0.0865 ± 0.0009	0.0868 ± 0.0015	0.0897 ± 0.0011	0.0937 ± 0.0009
	Water Uptake	12.987 ± 0.001	24.819 ± 0.091	25.252 ± 0.089	29.437 ± 0.088	35.209 ± 0.093
N438 + Ag	W_wet_ (gm)	0.0765 ± 0.0011	0.0795 ± 0.0014	0.0842 ± 0.0009	0.0921 ± 0.0098	0.0988 ± 0.0015
	Water Uptake	17.331 ± 0.005	21.932 ± 0.003	29.141 ± 0.023	41.257 ± 0.015	51.533 ± 0.012
N438 + Ag + CNT (0.01)	W_wet_ (gm)	0.0645 ± 0.0016	0.0654 ± 0.0009	0.0760 ± 0.0098	0.0843 ± 0.0013	0.0916 ± 0.0018
	Water Uptake	18.566 ± 0.007	20.220 ± 0.002	39.705 ± 0.008	54.963 ± 0.012	68.382 ± 0.009
N438 + Ag + CNT (0.05)	W_wet_ (gm)	0.0780 ± 0.0017	0.0880 ± 0.0006	0.0914 ± 0.0089	0.0932 ± 0.0005	0.0953 ± 0.0002
	Water Uptake	16.591 ± 0.002	31.539 ± 0.004	36.621 ± 0.013	64.312 ± 0.078	76.334 ± 0.009
N438 + Ag + Graphene (0.01)	W_wet_ (gm)	0.0792 ± 0.0023	0.0809 ± 0.0015	0.0885 ± 0.0009	0.1017 ± 0.0001	0.1132 ± 0.0014
	Water Uptake	15.959 ± 0.001	18.448 ± 0.013	29.575 ± 0.016	48.909 ± 0.009	65.739 ± 0.002
N438 + Ag + Graphene (0.05)	W_wet_ (gm)	0.0857 ± 0.0015	0.0869 ± 0.0004	0.1148 ± 0.0085	0.1234 ± 0.0091	0.1359 ± 0.0051
	Water Uptake	18.862 ± 0.012	20.527 ± 0.003	59.223 ± 0.001	71.151 ± 0.009	88.488 ± 0.007

**Table 5:** Tip deflection of the IPMC membranes at different input voltages.

Input Voltage (V)	0.6	1.2	1.8	2
N438 + Ag	3.3 mm ± 0.02	7.5 mm ± 0.09	8.1 mm ± 0.05	8.8 mm ± 0.01
N438 + Ag + CNT (0.01)	3.5 mm ± 0.03	8.4 mm ± 0.10	8.9 mm ± 0.07	9.2 mm ± 0.11
N438 + Ag + CNT (0.05)	4.3 mm ± 0.12	8.6 mm ± 0.05	10.2 mm ± 0.01	10.9 mm ± 0.08
N438 + Ag + Graphene (0.01)	3.9 mm ± 0.07	8.1 mm ± 0.11	8.8 mm ± 0.09	9.6 mm ± 0.02
N438 + Ag + Graphene (0.05)	4.2 mm ± 0.03	9.3 mm ± 0.05	11.6 mm ± 0.07	12.1 mm ± 0.01

**Table 6:** Tip force of the IPMC membranes at different input voltages.

Input Voltage (V)	0.6	1.2	1.8	2
N438 + Ag	1.85 mN	1.93 mN	1.95 mN	1.96 mN
N438 + Ag + CNT (0.01)	2.10 mN	2.63 mN	2.66 mN	2.73 mN
N438 + Ag + CNT (0.05)	2.17 mN	2.82 mN	2.81 mN	2.83 mN
N438 + Ag + Graphene (0.01)	2.05 mN	2.64 mN	2.73 mN	2.74 mN
N438 + Ag + Graphene (0.05)	2.30 mN	3.10 mN	3.17 mN	3.18 mN

## Data Availability

The data presented in this study are available on request from the corresponding author.
